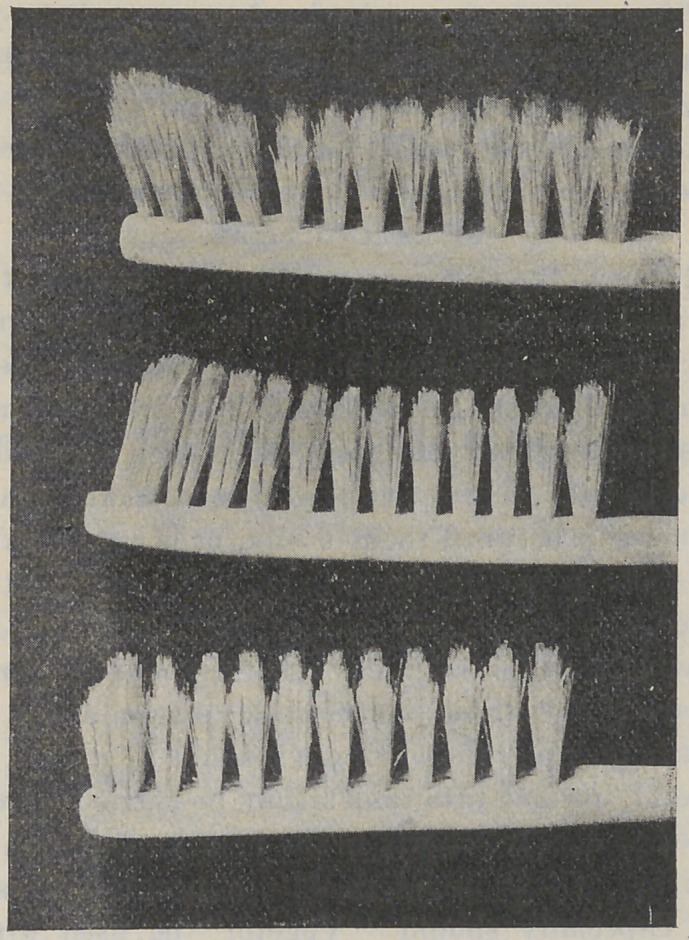# The Civilized Mouth

**Published:** 1899-11

**Authors:** 


### THE DENTAL REGISTER.

VOL. LIII.] NOVEMBER, 1899. [No. 11.

### COMMUNICATIONS.

### The Civilized Mouth.

Nine-tenths of the disease germs which enter the system are taken in through the mouth, therefore, no part of the person needs greater care.

With every step toward higher civilization comes its relative demand for closer attention to the physical being, and this should begin at the "vestibule" where the greatest danger lies. First, in what enters; second, the condition of the "vestibule" itself.

It is an accepted fact, that the mouth is constantly inhabited by numerous germs, many of which are disease producing. Within the short space of an hour, the particles of food remaining in the mouth become filled with microbes, causing bad breath, and irritating the soft tissues.

"Vegetol" is the result of an earnest effort to combat the evil effects of these germs. It has been so named from its prophylactic (preventive) and remedial action.

The word "Vegetol" is derived from the Latin word, *Vegetus*, which means brisk, active, vigorous, and "Ol"—meaning oil, and is given to this new combination, because of its vigorous, destructive action upon all kinds of bacteria, and its softening and healing effect upon the skin and mucous membranes. The name also indicates its vegetable origin.

"Vegetol" is a combination of vegetable matter and mild chemical compounds. The vegetable part of this composition is taken from the hard portions of cereals, and is prepared especially for the mechanical effect it has upon the teeth and gums in removing any foreign substance. Being much coarser than ordinary tooth powder, it cleanses more effectively. Anything which is immovable by the use of "Vegetol" should have the attention of a dentist.

The chemical parts of "Vegetol" are composed of sodium borate and potassium-chlorate, and are so combined and associated with the vegetable base, that when they come into contact with the saliva, they are dissolved by it, forming a pleasant fluid. This fluid arrests fermentation and putrefaction by destroying the bacteria (germs).

Lactic acid is formed in the mouth by the action of bacteria upon all cooked starches, and is known to be the great cause of decay of the teeth.

"Vegetol" being a germicide and antiseptic, not only cleanses the teeth mechanically, but penetrates the smallest irregularities in the teeth and gums, neutralizing the lactic acid of this ferment.

Saliva freighted with microbes, being unconsciously swallowed, often produces fermentation in the stomach, which results in eructations, sour stomach, headache, etc.

A small portion of "Vegetol" allowed to dissolve in the saliva, and swallowed, will quickly correct this disorder.

Normal saliva is alkaline, and should remain as nature intended; in a healthy mouth it is never acid unless immediately after eating acid foods—like fruits, vegetables, pickles, etc. The alkalinity of normal saliva being weak, few mouths are capable of recovery from an acid condition before more food is taken.

It is not possible, however, to be in perfect health and avoid acids, which, unfortunately, assist in the destruction of our teeth. But a small portion of "Vegetol" taken into the mouth after eating will restore it to an alkaline condition, counteracting these ill effects.

Decay of the teeth is arrested, or at least proceeds very slowly, when the saliva is normally alkaline.

It is due to the alkaline saliva of animals that their teeth do not decay. The exception to this rule is found in domestic animals, or those kept as pets or in confinement, and fed upon prepared foods.

The saliva of cattle, sheep, dogs, rabbits, etc., is essentially strong in alkaline reaction, and this accounts for their perfect digestion, as well as their immunity from decayed teeth. This is not true of mankind, and in order to compensate for the physical defects of civilization, caused by eating prepared foods, the mouth should have conscious and continual attention. Perfect health seems to demand that the saliva be artificially restored to its normal alkalinity.

It would seem equally rational to make conscious effort to keep the fluids of the mouth in a condition to preserve the teeth and aid digestion, as to wear clothing for warmth, or glasses to assist the eyes.

### AS A MEDICINE

"Vegetol" may be taken into the stomach, the same as in the mouth, to correct abnormal conditions, since the physiology of these two organs is very closely related.

The little spoon in the "Vegetol" box holds, when full, about five grains, and the tablets contain the same amount. This quantity can be taken with great relief and benefit in cases of fermentive or putrefactive indigestion. If one or two doses do not give relief, it may be repeated at intervals of one-half hour, if necessary; but one dose is usually sufficient.

### MOUTH WASHES.

The mucus, of which the saliva is largely composed, does not dissolve in water, ether or alcohol, but dissolves easily in alkaline solutions.

Of the many liquid preparations on the market which claim to sterilize and purify the mouth, it is found by scientific analysis that only one out of the great number has more than a slight destructive effect upon bacteria, failing of their object on account of their lack of alkalinity. Any attempt to wash the saliva from the cavities in and about the teeth with anything but an alkaline solution, is much like trying to remove grease without soap—the undisturbed portions of saliva remaining filled with micro-organisms.

Saliva is the fittest fluid for the mouth, because nature supplies it. "Vegetol" is soluble in saliva, and furthermore sustains its alkalinity. The saliva is already in the places where the damage to the teeth is being done, and by utilizing it to dissolve "Vegetol," an antiseptic wash is produced, the mouth is kept normal and nature's means of protection, or prophylaxis, is unimpaired.

Besides being an antiseptic wash, "Vegetol" possesses great healing properties for ulcerated mouth or throat. It not only heals, but prevents ulcerations and sore gums.

### TOOTH POWDER.

Unlike all other dentifrices or tooth powders, "Vegetol" contains no material which can possibly injure the teeth, even if used excessively. The chalk or ground sea shells, which compose the bulk of other tooth powders, if coarse enough to really cleanse the teeth, and used enough to keep them so, must injure them. The cereal in "Vegetol" is the vegetable substitute for this earthy matter.

The natural polish of the teeth is perfect, and if kept free from deposits they can not be improved in color by the use of pomace or any gritty substance.

### SOAP.

As a dentifrice and mouth wash, soap fails of its object; if mixed with powder it merely lubricates each particle of it, and the bits of food about the teeth, causing the brush to slip easily over instead of removing them.

Soap relieves the mouth of oils and mucus, as it will the skin, but does not remove the tartar nor destroy the bacteria. Toilet soaps, as a rule, are not strongly alkaline; therefore can not neutralize the acids of decay to any great degree.

There can be no serious objection to the use of pure soap in the mouth, but it can not be relied upon to cleanse or purify sufficiently.

### THE BREATH.

It is an erroneous idea that the breath is contaminated by a foul stomach.

There are certain foods which affect the breath, such as onions, garlic, etc., but their odors come either from particles remaining in the mouth, or are exhaled by the lungs from the blood.

The breath becomes contaminated only through the surfaces with which it comes in contact, viz: The nose and its accessory cavities, the mouth, the throat, the bronchial tubes and the lungs. Of all these surfaces that of the mouth is most frequently at fault. To verify this statement, close the mouth and test the breath as it comes from the nostrils.

It should be borne in mind that a sweet breath is almost sure to follow if the mouth is kept free from putrefaction.

### TARTAR.

Tartar was so called by the ancients from its fancied resemblance to the hard deposits of cream of tartar found on the inside of old wine casks. It is composed of bone phosphate and is a precipitate from saliva. It will adhere to artificial teeth or bridge-work as upon the natural teeth, becoming at times very hard if undisturbed. In this condition it can be removed only by the use of steel instruments.

It is often asked why tartar can not be removed by chemicals. The answer is that the teeth and tartar are of much the same chemical composition. Hence anything that will act upon tartar, also injures the teeth, the difference being that the teeth are harder and would not dissolve so rapidly.

Tartar does not cause the teeth to decay, but occasions their loss more effectually by inducing destruction of the bone which supports them. This result comes by the deposit of tartar about the necks of the teeth and under the margins of the gums where the latter do not adhere to the teeth. As this hard deposit accumulates, the gums become inflamed and sore. Then, when the teeth are brushed the inflamed gums are rubbed against this rough deposit causing them to bleed. The bleeding is an effort on the part of nature to repair damage, and bone being soluble by this process, the thin plates about the teeth are finally destroyed, and the teeth either drop out or may be removed with the fingers. The only way to prevent this misfortune in later years is to keep the tartar from accumulating in early life.

It is a fact that most teeth which are lost in this way have no decay.

It is also a fact that there is no disease of the mouth more under the control of the person than this one, if taken in time; but if allowed to go too far, no disease of the teeth so baffles the skill of the dentist.

"Bleeding gums" indicate hard deposits about the teeth, and demand the attention of a dentist.

The teeth and gums should have a thorough cleansing at least twice every day with brush, pick and "Vegetol," and if they have a great tendency to tartar or decay, it should be done oftener. This care, taken for general health and cleanliness, produces one of the most attractive features of the person. When the lips and gums are of a healthy red and pink, the teeth pure and strong, accompanied by a sweet breath, the mouth indicates health and refinement.

BRUSHES.

Economy as well as utility demand that the quality of material be good, but most important is the manner in which the bristles of the brush are cut. No brush is efficient in which the bristles are all of the same length, for the reason that they then brush only the exposed parts of the teeth. By the use of a serrated brush (see illustrations) the longer bristles reach to some distance between the teeth, and into the depressions of their crowns, and remove particles of food and soft tartar which serve as hot-beds for germs.

The bristles of the brush should be stiff; and at least one-half the width of the brush should be allowed to rub the gums, insuring the removal of the food and tartar about the necks of the teeth. By this massage treatment of the gums, they adhere closely about the teeth and become firm and healthy.

SELECTING A BRUSH.

These cuts represent various forms and sizes of serrated brushes, any one of which will be efficient. The object of the pictures is to enable one to select from a stock of brushes those which will do the work best, for the majority of brushes are of little use.

CARE OF BRUSHES.

It will be observed that the rows of the bristles of all these brushes are widely separated; this is necessary in keeping the brush sweet and clean, by admitting air between the bristles, and permitting the easy removal of lodgments.

The bristles in a poor brush soon mat down, retaining moisture, fostering putrefaction and microbes.

After using, the brush should be well washed, rubbed with a dry towel, and stood with handle down in vase or rack; this treatment is well worth while, for a dry brush is always pure, cleanses better, and its durability is greatly increased.

PICKS AND FLOSS.

In addition to the stiff serrated brush, soft wood tooth-picks should be used between the teeth to insure the removal of food and tartar—the soft wood rubbing and cleansing the adjacent sides of the teeth where the bristles of the brush do not reach.

Where the crowns of the teeth touch each other silk or linen floss should be used, but not twisted thread of any kind. If the floss does not slip between them easily, or is cut or ravelled it is evidence of decay or defective fillings, and your dentist should be consulted.

"Vegetol" tooth powder has been compounded for the purpose of caring for these inaccessible places about the teeth which brush, pick and floss will not reach.

"Vegetol" formed into tablets may be carried in the pocket and allowed to dissolve slowly in the mouth after each meal and before retiring, if it is impracticable to use the brush so frequently. Especially should "Vegetol" be used after eating acid foods.

HOW TO USE "VEGETOL."

The spoon in the box is for the purpose of measuring the amount to be placed upon the brush, or in the palm of the hand. The latter method is convenient and prevents dipping the damp brush into the powder. The spoon holds, ordinarily, the amount one should use. However, there is no danger from using too much. In order to remove deposits any dentifrice must be used very freely.

"Vegetol" dentifrice, when first used may cause a tingling or smarting, and in rare cases a little soreness for a short time, but this particular feature hardens and strengthens the gums by continued use.

"VEGETOL" TABLETS.

"Vegetol" is made into five grain tablets, which can be carried in the pocket or shopping-bag. Some prefer this form to powder, but our intention is to supply "Vegetol" in convenient form for those who are troubled with indigestion, or with acid mouth after eating sweets, fruits or salads. These tablets will dissolve in the mouth readily, and are identical with the powder except in form.

FOR BAD BREATH.

A tablet allowed to dissolve slowly in the mouth will remove disagreeable odors of any kind, and leave in its place a refreshing taste and sweet breath.

## Figures and Tables

**Figure f1:**
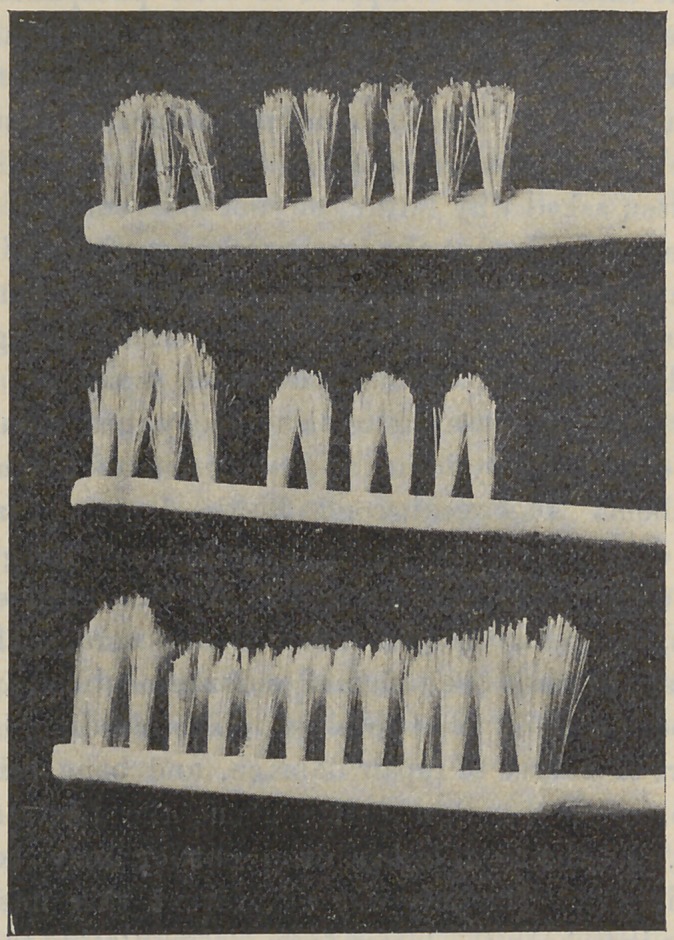


**Figure f2:**